# The Role of Extracellular Vesicles in the Development and Treatment of Psoriasis: Narrative Review

**DOI:** 10.3390/pharmaceutics16121586

**Published:** 2024-12-12

**Authors:** Bin Tang, Yang Bi, Xuwei Zheng, Yujie Yang, Xiaobing Huang, Kexin Yang, Haixin Zhong, Ling Han, Chuanjian Lu, Haiming Chen

**Affiliations:** 1 The Second Clinical College of Guangzhou University of Chinese Medicine, Guangzhou 510120, China; 2 State Key Laboratory of Dampness Syndrome of Chinese Medicine, The Second Affiliated Hospital of Guangzhou University of Chinese Medicine (Guangdong Provincial Hospital of Chinese Medicine), Guangzhou 510120, China; 3 Guangdong Provincial Clinical Medicine Research Center for Chinese Medicine Dermatology, Guangzhou 510120, China; 4 Guangdong-Hongkong-Macau Joint Lab on Chinese Medicine and Immune Disease Research, Guangzhou University of Chinese Medicine, Guangzhou 510120, China; 5 Shenzhen Hospital (Futian) of Guangzhou University of Chinese Medicine, Shenzhen 518000, China; 6 Hospital of Osteopathy The Third Affiliated Hospital of Guangzhou University of Chinese Medicine, Guangzhou 510378, China

**Keywords:** extracellular vesicle, psoriasis, therapy, vesicle cargo, nano particles

## Abstract

Psoriasis is a chronic inflammatory polygenic disease with significant impacts on skin and joints, leading to substantial treatment challenges and healthcare costs. The quest for novel therapeutic avenues has recently highlighted extracellular vesicles (EVs) due to their potential as biomarkers and therapeutic agents in autoimmune diseases, including psoriasis. EVs are nano-sized, lipid membrane-bound particles secreted by cells that have emerged as promising tools for targeted drug delivery, owing to their unique structure. This review delves into how EVs, either as mediators of cell communication or via their cargo (such as miRNA), directly participate in the pathology of psoriasis, influencing processes such as immune regulation, cell proliferation, and differentiation. Furthermore, this review explores the innovative application of EVs in psoriasis treatment, both as direct therapeutic agents and as vehicles for drug delivery, offering a novel approach to overcoming the current treatment limitations.

## 1. Introduction

Psoriasis is a chronic, non-infectious, polygenic hereditary inflammatory disease associated with autoreactive immune responses. This condition can cause recurrent episodes of skin and joint inflammation, leading to significant discomfort and a reduction in quality of life [1]. The incidence of psoriasis is generally higher in adults than in children, and its geographical distribution is uneven [2]. Multiple studies have reported that the age of onset in female patients is typically earlier than in males [3]. Additionally, patients with psoriasis are at an increased risk of developing several comorbidities, including metabolic syndrome, cardiovascular diseases [4], mental health issues, psoriatic arthritis, and other immune-mediated diseases [5]. Both severe and mild forms of psoriasis are associated with an increased risk of stroke and myocardial infarction [6]. 

Psoriasis is primarily driven by the interplay of genetic predispositions, environmental triggers [7], and aberrant immune responses. Despite extensive research, the precise pathomechanism underlying psoriasis remains elusive. Notably, over the past two decades, the IL-23/IL-17 axis has been recognized as a pivotal element in the pathogenesis of this immune-mediated inflammatory condition [8]. A myriad of biologic agents targeting the IL-23 and IL-17 pathways have been developed and shown remarkable efficacy in psoriasis management [9]. Additionally, pro-inflammatory cytokines like TNF-α, IL-23, and IL-17, synthesized by immune cells, are implicated in the pathologic alterations characteristic of psoriasis [10]. Recent research has increasingly focused on the interplay between mesenchymal stem cells (MSCs) and psoriasis. Under the influence of growth factors and inflammatory mediators, naive CD4+ cells differentiate into various subtypes including Th1, Th2, and Th17. MSCs derived from psoriatic skin have been observed to upregulate Th1 and Th17 cells, highlighting a dysregulated Th1–Th17/Th2 axis in psoriasis [11]. Emerging evidence suggests that oxidative stress, driven by an excess of reactive oxygen species (ROS), plays a significant role in psoriasis pathogenesis [12]. Intriguingly, human adipose tissue-derived mesenchymal stem cells (hAD-MSCs) have demonstrated the ability to inhibit ROS production, indicating a potential therapeutic role in modulating ROS levels in psoriasis treatment [13]. Given the recurrent, complicated, and often refractory nature of psoriasis, extensive research is ongoing to optimize treatment strategies. Current therapeutic regimens include methotrexate, cyclosporine, acitretin, imuran, leflunomide, mycophenolate, glucocorticoids, antibiotics, and biologics. For instance, ixekizumab is now utilized in managing erythrodermic psoriasis (EP) [14]. Other IL-17 antagonists, such as secukinumab [15] and brodalumab [16], have also demonstrated effectiveness against EP. Topical agents like acitretin are commonly considered first-line treatments for psoriatic lesions in clinical settings. 

Recent comprehensive research indicates that certain pharmacological interventions for psoriasis are associated with a spectrum of adverse events. These include the potential onset of multiple sclerosis [17], gastrointestinal complications, such as bleeding and ulceration [18], hepatic dysfunction, and respiratory tract infections [19]. Additionally, there are concerns about dyslipidemia, hypertension, and neurological disorders as side effects. Notably, studies have highlighted that the use of acitretin may lead to adverse reproductive outcomes, such as abortion in adolescent females [20]. Furthermore, the absence of definitive diagnostic biomarkers poses significant challenges in the early detection and management of psoriasis, underscoring the urgency for innovative therapeutic approaches. Consequently, there is a pressing need to develop novel strategies that effectively treat psoriasis while minimizing the risk of these adverse events. 

Extracellular vesicles (EVs), encompassing exosomes, microvesicles, and apoptotic bodies, are nanoscale particles originating from cellular membranes and have emerged as a significant resource for the identification of biomarkers in various diseases, including psoriasis [21]. EVs are detectable in a myriad of body fluids, such as blood [22], urine [23], amniotic fluid [24], cerebrospinal fluid, breast milk, saliva [25], and synovial fluid [26]. They play a crucial role in immune modulation, including suppression, adhesion, and antigen presentation. Notably, alterations in plasma lipid profiles in psoriasis patients have been correlated with EVs, suggesting their potential utility as biomarkers for psoriasis diagnosis and for monitoring therapeutic responses [27]. Recent advancements have underscored the growing importance of EVs in the management of numerous diseases, extending to rheumatoid arthritis [28], retinal disorders [29], vitiligo [30], chronic wounds [31], autoimmune diseases [32], and coronary artery disease [33]. Given the autoimmune nature of psoriasis, the interplay between EVs and psoriasis treatment warrants in-depth exploration. This burgeoning area of research holds promise for enhancing our understanding of psoriasis pathophysiology and for the development of innovative therapeutic strategies.

In this comprehensive review, we delve into the role of EVs in psoriasis. To ensure a comprehensive and focused discussion, we performed a systematic literature search using databases such as PubMed, Embase, and Web of Science. The search strategy combined relevant keywords and MeSH terms, including “extracellular vesicles”, “psoriasis”, “pathogenesis”, and “treatment”. Studies were included if they were peer-reviewed, directly addressed the role of extracellular vesicles in psoriasis pathogenesis or treatment and were published in English. Exclusion criteria encompassed non-original research, studies with incomplete data, or those deemed outside the scope of this review. This approach was implemented to provide a comprehensive and scientifically rigorous foundation for the discussion. We explored the potential of EVs as biomarkers and therapeutic agents in autoimmune conditions, focusing on their unique capability for targeted drug delivery. We also discussed the application of EVs in skin-related therapies, examining their involvement in the immune response, cell proliferation, and differentiation processes in psoriasis. This review also highlights the dual utility of EVs as direct therapeutic agents and as vehicles for drug delivery, offering a promising new avenue in psoriasis treatment.

## 2. Characterization and Analysis of Extracellular Vesicles: Subtypes, Biogenesis, Functional Roles, Isolation Techniques, and Identification Methods

Cellular communication and the evolution of biological systems are significantly facilitated by the release of vesicles from cellular membranes into the extracellular space. The initial discovery of these vesicles dates back to the 1940s when Chargaff E and West R observed a reduction in clotting times upon adding a sub-cellular fraction isolated from plasma [34], suggesting that cell-free plasma components expedite clotting processes. Subsequent research, approximately two decades later, identified these sub-cellular elements as small vesicles, later termed ‘platelet dust’ [35]. Concurrently, the concept of exosomes emerged with the identification of ‘exfoliated membrane vesicles’ possessing potential physiological functions [36]. Exosomes, ascertained to be derived from plasma membranes, have garnered continuous scientific interest, particularly in their role as potential biomarkers for diseases such as cancer, rheumatoid arthritis, and psoriasis. The publication of comprehensive guidelines by the International Society of Extracellular Vesicles (ISEV) in 2018 marked a significant advancement in our understanding of EVs. These guidelines are now considered the gold standard in EV research, providing a framework to enhance our comprehension of the intricate relationship between EVs and psoriasis.

In recent times, nanobiological therapeutics have emerged as a groundbreaking approach in the treatment of a multitude of diseases, including psoriasis. Distinguished from traditional exogenous medications, EVs represent an endogenous modality, either serving as vehicles for drug delivery or acting as therapeutic agents themselves. Characterized by their convenience, safety profile, and reduced incidence of adverse effects, EVs hold substantial promise in advancing the treatment of psoriasis. It is imperative to intensify research efforts focusing on the genesis, characterization, and application of EVs, to harness their full therapeutic potential in the management of psoriasis.

### 2.1. Subtype and Biogenesis

EVs are predominantly secreted by cells in a quiescent state, playing a vital role in enhancing intercellular communication, particularly during cellular proliferation. The release of EVs is notably augmented when cells encounter activation or stress conditions, such as exposure to cytokines and proinflammatory stimuli, leading to a significant increase in the production of EVs. Understanding the mechanisms underlying the generation of various EV subtypes is critical. This knowledge is pivotal not only for the improved identification and isolation of EVs but for their potential application in clinical settings as biomarkers and vehicles for targeted drug delivery in the treatment of psoriasis. 

**Figure 1:**
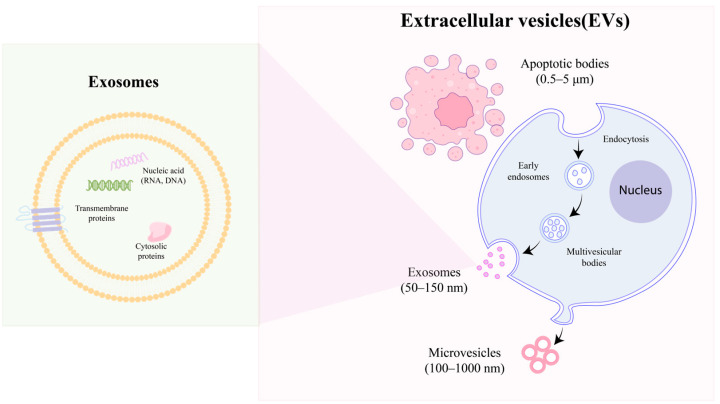
Biogenesis of EVs: microvesicles, exosomes, and apoptotic bodies.

EVs present a diverse and complex array of subtypes within the biosphere, leading to various classification methodologies. Predominantly, EVs are categorized based on their size and biogenesis. Regarding their generation, EVs are broadly divided into three distinct subtypes: exosomes, microvesicles, and apoptotic bodies, as illustrated in Figure 1. The International Society for Extracellular Vesicles (ISEV) classifies exosomes, which range in size from 50 to 150 nm, as small EVs (sEVs). These ‘classical’ exosomes originate from the endocytic pathway, undergoing a unique ‘double invagination and release’ process at the plasma membrane. This process initiates with the plasma membrane forming a cup-shaped invagination, leading to the creation of early-sorting endosomes [37]. These endosomes subsequently evolve into late-sorting endosomes (LSEs), eventually giving rise to multivesicular bodies (MVBs) and, finally, exosomes. The secondary invagination occurs within the MVBs, involving the endocytosis of the endosomal limiting membrane. This explains the presence of multiple LSEs within a single MVB. The release of exosomes occurs when the contents of the MVBs are expelled into the extracellular space.

Distinctly larger than exosomes, microvesicles typically measure between 250 and 400 nm in diameter and are categorized as medium-sized EVs [38]. In contrast to exosomes, microvesicles are exclusively released via exocytosis, signifying a direct shedding from the cellular membrane. It is important to note that, while the budding of microvesicles from the biological membrane is a stochastic process, their site of release is highly specific [39]. This process is influenced by the redistribution of phospholipids and the action of Rho–kinase-mediated myosin light chain phosphorylation, which together facilitate the contractile machinery essential for the vesicle’s extrusion and subsequent detachment [40].

Apoptotic cell-derived EVs (ApoEVs), the largest category of extracellular vesicles, typically range in size from approximately 1 to 5 μm and originate from cells undergoing apoptosis. Within this category, two subtypes are distinguished: the larger subset known as apoptotic bodies (ApoBDs), which are membrane-bound vesicles [41], and a smaller subset, termed apoptotic microvesicles (ApoMVs) [42]. The process of apoptotic cell decomposition is characterized by four primary stages [43]. Initially, the cell exhibits lamellipodia at its periphery. Subsequently, blebs begin to bud from the plasma membrane, leading to significant alterations in cell morphology driven by dynamic blebbing. This stage is followed by the formation of a thin membrane structure. Ultimately, ApoBDs dissociate from the main apoptotic cell bodies, marking the final stage of this process.

### 2.2. Function

EVs are minuscule entities that encapsulate a diverse array of bioactive molecules, ubiquitously present both in vivo within humans and across various micro-environments. The functional scope of EVs is extensive and multifaceted. Initially perceived merely as vehicles for the disposal of cellular debris [44], exosomes have since been acknowledged for their critical roles in intercellular communication, immune modulation, antigen presentation, and the conveyance of signaling molecules. EVs are capable of transferring proteins, mRNA, microRNA, and lipids to both proximal and remote target cells, thereby facilitating cellular activation and reprogramming of cellular functions [45]. The interaction between EVs and recipient cells occurs through three primary mechanisms: ligand–receptor interactions, membrane integration, and endocytosis [46].

Owing to their distinct antigenic profiles, EVs have garnered significant interest as potential biomarkers for a range of diseases. For instance, the presence of EVs in the cerebrospinal fluid of Alzheimer’s disease patients has been demonstrated to serve as a reliable biomarker for this condition [47]. Furthermore, EVs offer promising prospects in disease prognostication. For example, urinary EVs have been shown to be predictive of liver injury [48]. In the context of psoriatic arthritis (PsA), exosomes have been identified as contributors to the enhanced formation of osteoclasts, a phenomenon observed independently of variables such as age, disease activity, and erythrocyte sedimentation rates [49].

### 2.3. Isolation and Identification

EVs are present in a variety of biological fluids, including tears, amniotic fluid, cerebrospinal fluid, and blood. The isolation of EVs from these fluids involves diverse methodologies. Historically, ultracentrifugation has been considered the gold standard for EV isolation, and it continues to be employed as the primary method in numerous studies, such as those exploring the pathological relationship between exosomal miRNA and psoriasis. This technique entails a series of steps: the removal of cells, cell debris, and larger vesicles, followed by the precipitation and subsequent collection of EVs. However, the use of ultracentrifugation presents several drawbacks, including its time-intensive nature, requiring upwards of 30 h, and the co-isolation of impurities [49].

Given these limitations, the development of novel and more efficient isolation methods is crucial. Over time, a range of techniques has been devised to isolate EVs or to combine different methods for obtaining monodisperse EVs. Notably, in 2022, size-exclusion chromatography emerged as a promising approach for the isolation of EVs in patients with psoriasis.

The post-isolation identification of EVs is a critical component in their application for disease treatment research. The initial step involves the morphological assessment of EVs. Transmission electron microscopy (TEM) is utilized to observe the bilayer phospholipid structure characteristic of EVs. Additionally, the grain density distribution serves as an indicator for the presence of EVs, as delineated by the International Society for Extracellular Vesicles (ISEV). For particle size analysis, nanoparticle tracking analysis (NTA) is highly recommended, offering the capability to select particles around 100 nm with notable speed and precision.

Furthermore, the detection of specific biomarkers on EVs is essential for their identification. Western Blot analysis can be employed to detect typical markers present in plasma samples, such as CD9, CD63, CD81, and TSG101 [50]. These markers are instrumental in confirming the presence and characterizing the nature of EVs.

## 3. Investigating the Role of Extracellular Vesicles in the Physiology and Pathophysiology of Skin and Its Appendages

Acknowledged to be the largest organ of the human body, the skin constitutes approximately 16 percent of total body weight, underscoring the importance of comprehending its intricate structure. Anatomically, the skin is stratified into three primary layers: the hypodermis, dermis, and epidermis. The epidermal layer itself is further divided into five sublayers: the stratum corneum, stratum lucidum, stratum granulosum, stratum spinosum, and stratum basale. Functioning as a critical barrier, the skin provides resistance against external insults, including thermal and mechanical stress, thereby safeguarding the body [51]. Given its extensive surface area and the significant alterations it undergoes in various diseases, such as psoriasis, investigating the interplay between EVs and the physiological as well as pathological processes of the skin is of paramount importance.

### 3.1. The Involvement in Cutaneous Immunity in Sweat Exosomes

Sweat, a secretion of the skin, is a transparent, clear, and hypotonic biological fluid. It has gained recognition for its clinical significance in the diagnosis of conditions such as hyperhidrosis [52]. While the presence of exosomes has been documented in various bodily fluids, their occurrence in sweat has been less frequently reported [53]. Notably, exosomal content in sweat has been identified to be devoid of contaminants, such as bacteria, corneocytes, or holocrine secretions from sebaceous glands. Consequently, sweat is considered to be a superior biofluid for proteomic analysis, offering a relatively uncontaminated medium compared to other bodily fluids [54]. In a seminal study conducted in 2018, Wu et al. elucidated the potential role of exosomal sweat in antimicrobial activity and the maintenance of cutaneous immune homeostasis. This investigation, which analyzed sweat samples from healthy adult volunteers post-aerobic exercise, suggested a functional significance of sweat-derived exosomes in skin physiology [53]. Subsequently, in 2020, Karvinen et al. reported a pioneering finding: an increase in miR-21 levels within sweat EVs following aerobic threshold tests. This discovery not only confirmed the presence of miRNA within sweat EVs but extended its known existence beyond serum [55]. These findings collectively underscore the potential research value of focusing on exosomal sweat as a novel biological specimen for investigative studies in cutaneous and systemic physiology.

### 3.2. Enhanced the Cutaneous Regenerative via Extracellular Vesicle

In addition to their role in sweat composition, EVs have been increasingly recognized for their significant contribution to cutaneous regeneration. Normal skin healing encompasses four stages: hemostasis, inflammation, proliferation, and remodeling [56]. Emerging research has highlighted the critical involvement of EVs in key processes, such as cellular proliferation, migration [57], and collagen deposition [58]. The mechanism of action involves MSC-derived extracellular vesicles containing transcriptionally active STAT3, which activate AKT, ERK1/2, and STAT3 pathways and induce the expression of various growth factors. This process enhances the growth and migration of fibroblasts in chronic wounds and promotes angiogenesis in vitro. Moreover, the activation and enhancement of signaling pathways like AKT/ERK and Wnt, mediated by EVs, have been implicated in promoting skin wound closure. These findings indicate the potential of EVs in facilitating accelerated wound healing and reducing scar formation. 

Despite the growing intensity of research in this area, the application of EVs as a reliable therapeutic modality is still in its nascent stages. A notable challenge is the lack of standardized protocols across different studies, which impedes the establishment of definitive clinical guidelines. Hence, the efficacy of EVs in wound healing warrants further investigation. Additionally, the impact of EVs on hair growth, particularly in patients with psoriasis, remains an area requiring more in-depth exploration [59].

### 3.3. Exploring the Application of Keratinocyte-Derived Extracellular Vesicles for the Protection of Hair Follicles

In recent years, there has been a growing body of evidence supporting the active involvement of exogenous apoptotic cell-derived extracellular vesicles (apoEVs) in hair follicle metabolism. This has led to increased research interest in the interaction between EVs and hair follicles [60]. Notably, exosomes secreted by stem cells have been demonstrated to effectively promote hair follicle regeneration [61]. Additionally, recent findings suggest that exosomes derived from adipose stem cells hold therapeutic potential for the treatment of autoimmune diseases [62]. 

The role of keratinocyte proliferation in the pathogenesis of psoriasis is well-established. The facilitation of intercellular communication by extracellular vesicles has sparked significant interest in the scientific community, particularly regarding the crosstalk between keratinocytes and melanocytes. Exosomes secreted by keratinocytes, through a paracrine mechanism, have been shown to regulate skin pigmentation [63]. In 2011, studies highlighted that certain pro-inflammatory factors, including IL-1α, which are associated with psoriatic skin, might impede melanogenesis [64]. Subsequent research in 2015 revealed that keratinocyte-derived exosomes enhance the expression and activity of melanosomal proteins, thereby augmenting melanin synthesis [65]. These exosomes have been identified as carriers of miRNAs that modulate pigmentation. Notably, in 2021, it was first demonstrated that exosomes from keratinocytes serve as a fundamental communicative bridge between keratinocytes and hair follicle stem cells (HFSCs) [66]. These discoveries have opened new avenues in exploring the relationship between keratinocyte-derived exosomes and psoriasis.

## 4. The Role of EVs in Psoriasis

**Figure 2:**
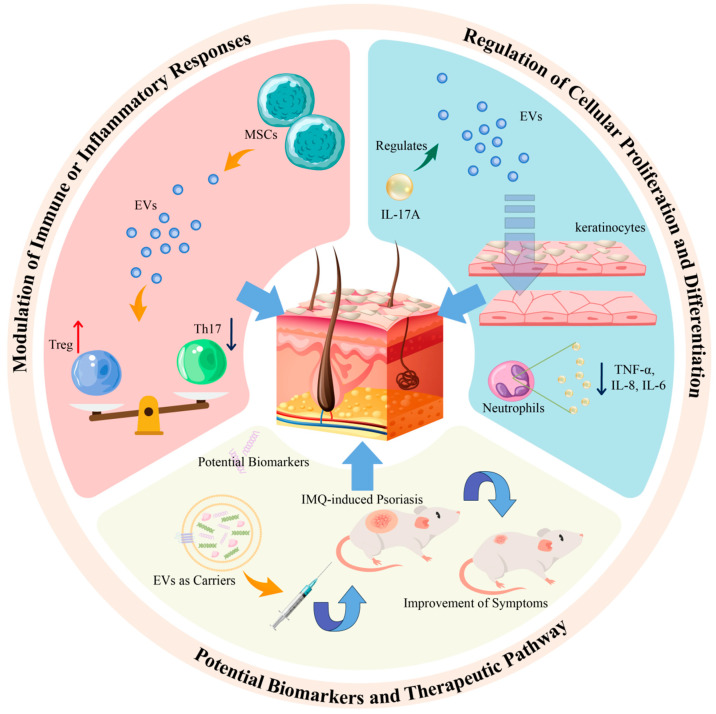
The role of EVs in psoriasis is divided into three parts: immunity, biomarkers, and treatment.

EVs have been identified as pivotal entities in targeting psoriasis, primarily through three distinct pathways: modulation of immune or inflammatory responses in psoriasis, regulation of cellular proliferation and differentiation, and the role of exosomal miRNA in the disease pathology (refer to Figure 2).

### 4.1. Exploring the Role of Extracellular Vesicles from Immune Cells in the Pathogenesis of Psoriasis

Th17 and Treg cells are two critical T cell subtypes with significant roles in autoimmune disorders, including psoriasis. Th17 cells are known for their secretion of IL-17, a cytokine that activates receptors in keratinocytes and is implicated in the pathogenesis of psoriasis, often in conjunction with cytokines secreted by Th1 and Th2 cells [67]. Conversely, Treg cells, which possess anti-inflammatory properties, exhibit suppressed activity in the peripheral blood of psoriasis patients. This suppression can be indirectly modulated by the anti-inflammatory effects of IL-10 [68,69]. A noted imbalance between Th17 and Treg cell levels has been correlated with the development of psoriasis. Intriguingly, recent studies have shown that exosomes derived from MSCs can mitigate the differentiation of T cells into Th17 cells while simultaneously augmenting Treg cell levels. This suggests that MSC-derived exosomes may influence the pathophysiology of psoriasis by modulating the equilibrium between Th17 and Treg cells, although the precise mechanisms underlying this regulation remain to be fully elucidated [70]. MSC-derived extracellular vesicles can suppress the expression of inflammatory cytokines TNF-α and IL-1β, induce the conversion of T helper type 1 cells to T helper type 2 cells, and reduce the differentiation of T cells into IL-17-producing effector T cells. Additionally, MSC-derived extracellular vesicles enhance the expression of cytotoxic T-lymphocyte-associated protein 4 (CTLA-4), a key protein associated with Treg cells.

It has been observed that the levels of inflammatory factors in blood samples from patients with psoriasis are significantly elevated compared to those in healthy individuals. This suggests that exosomes might serve as valuable biomarkers for assessing the treatment efficacy in psoriasis patients [71]. Consequently, there is a growing interest in exploring the expression of inflammatory mediators like IL-17A in the context of the relationship between EVs and psoriasis. Research has demonstrated that cytokines, such as IL-17A and IFN-γ, can enhance the secretion of EVs in HaCaT cells, indicating the potential role of EVs as integral components in the psoriasis-associated micro-environment [72]. Furthermore, exosomes derived from neutrophils have been shown to augment the levels of inflammatory factors, including IL-1β and TNF-α, through their endocytosis by keratinocytes. These inflammatory mediators are also pivotal in the pathogenesis of psoriasis. In psoriatic lesions, there is an observed increase in PLA2 activation. Notably, mast cell-derived exosomes, induced by IFN-α, have been found capable of transferring the activity of cytoplasmic PLA2 to adjacent CD1a-expressing cells, thereby stimulating the production of IL-22 and IL-17A [73].

### 4.2. Impact of Cytokine-Treated Keratinocyte-Derived Exosomes on Cell Proliferation in Psoriasis

EVs are ubiquitously released by virtually all normal cells throughout their lifecycle, with their cargo composition dynamically altering, especially in response to cytokine stimulation. Consequently, cytokine-induced EVs are implicated in aberrant cellular processes, including proliferation and differentiation, which are pivotal in pathological states. In psoriasis, the hyperproliferation of keratinocytes is a key pathological feature, suggesting that focusing on keratinocyte-derived EVs could yield deeper insights into the relationship between EVs and psoriasis. Research has demonstrated that IL-17A can modulate EV delivery, implying that EVs might potentiate the pro-inflammatory cascade in keratinocytes [74]. Intriguingly, studies have observed a reduction in the levels of IL-6, IL-8, and TNF-α in neutrophils following treatment with exosomes derived from keratinocytes.

### 4.3. Characterization of Extracellular Vesicle Cargo: Exploring Potential Biomarkers and Therapeutic Targets in Psoriasis

The lack of precise diagnostic biomarkers presents a significant challenge in the identification of effective treatments for psoriasis. Among potential biomarkers, microRNAs (miRNAs) have emerged as a key focus in psoriasis research. As the most extensively studied components within EVs, these noncoding, short regulatory RNAs are known to modulate the expression of a wide range of protein-coding genes, representing a predominant class of regulatory molecules [75]. A summary of recent findings on microRNAs present in EVs relevant to psoriasis is provided in Table 1.

Research has demonstrated distinct variations in microRNA profiles within EVs between T cells and their subcellular fractions. In healthy donors, the levels of miR-146a-5p, miR-150-5p, and miR-21-5p in exosomal microRNAs from Treg cells are notably higher compared to those in Th1–Th17 cells. Conversely, miR-106-5p, miR-155-5p, and miR-19a-3p in EVs originating from Th1/Th17 cells are considerably lower than in Treg-derived EVs. Interestingly, the treatment of psoriasis patients with Etanercept, which modulated some of these microRNAs, resulted in a significant decrease in the psoriasis area and severity index (PASI) [76]. Furthermore, among the miRNAs from serum-derived exosomes, miR-1305 and miR-6785-8p have been linked to the activation and differentiation of Th17 and Th1 cells, processes that are implicated in the mediation of psoriasis [77]. 

Recent studies have identified 36 co-expressed miRNAs across psoriatic arthritis (PsA), psoriasis vulgaris (PsV), rheumatoid arthritis (RA), and gouty arthritis (GA). Among these, five miRNAs—hsa-miR-151a-3p, hsa-miR-199a-5p, hsa-miR-370-3p, hsa-miR589-5p, and hsa-miR-769-5p—are believed to play a role in the shared pathologies of these diseases [50]. Notably, miR-199-5a has been shown to inhibit the expression of IL-6, and TNF-α is known to activate the ERK, P38, and JNK pathways [78]. Additionally, elevated levels of miR-381-3p in sEVs secreted by cytokine-treated keratinocytes have been reported, which contribute to the polarization of Th1 and Th17 cells, thereby exacerbating psoriasis progression [79]. Furthermore, miR-30e-5p operates through the Wnt pathway by modulating inflammation via its co-receptor, LRP6. These microRNAs are pivotal in the inflammation associated with psoriasis pathogenesis, particularly through the IL-6 and Wnt signaling pathways. Moreover, miR-199a-3p has been found to be significantly elevated in the serum EVs of psoriatic patients compared to healthy controls, and its levels have been observed to decrease following effective treatment [80].

In the realm of EV research, significant differences have been noted in the plasma EV content of patients with PsA compared to those with cutaneous-only psoriasis (PsC). Specifically, levels of let-7b-5p and miR-30e-5p were markedly reduced in PsA patients [81]. Let-7b, identified as an IL-6 ligand, has been found to exhibit elevated levels in PsA [82]. The challenge of differentiating psoriasis from pityriasis rosea (PR) has prompted investigations into the differential expression of exosomal microRNAs. Studies revealed that miR-500a-3p, miR-484, miR-185-5p, miR-27a-5p, and let-7f-5p were significantly lower in psoriatic patients and healthy individuals compared to PR patients, suggesting their potential as diagnostic markers for psoriasis and differentiation from PR. The miR-124-3p/FGFR2 axis has been implicated in ameliorating psoriasis-like inflammation by inhibiting keratinocyte proliferation and migration [83]. Further research suggests that miR-124-3p can alleviate the psoriatic micro-environment by targeting STAT3 and reducing IL-17A-induced inflammation. In vivo studies have demonstrated the efficacy of miR-124-3p delivered via EVs in mitigating discomfort in IMQ-induced mice, highlighting its therapeutic potential.

Furthermore, variations in miRNA levels in EVs are observed between different subtypes of psoriasis. For instance, hsa-miR-671-3p levels are significantly lower in PsA compared to PsV patients, underscoring the importance of distinguishing between psoriasis subtypes [21]. In addition to miRNA levels, target genes in these miRNAs, such as CREB1, RUNX2, and EGFR, exhibit variations in PsV and may contribute to the inflammatory response [84].

Beyond human-derived EVs, microbe-derived EVs are also being evaluated as potential biomarkers in psoriasis. Disparities in skin and gut bacterial microbiomes between psoriasis patients and healthy individuals have been noted, with severe psoriatic patients showing reduced levels of phyla Firmicutes and Fusobacteria. This microbiome variation may influence the composition of plasma-derived EVs [85]. Additionally, the biomarkers in EVs can vary based on their source, whether from blood plasma or serum. CD9, a common biomarker in EVs, has shown statistical significance in differentiating PsV from healthy controls, positioning it as a potential key biomarker in exosomal EVs for psoriasis.

**Table 1:** The role of exosomal microRNAs. Notes: ↓ represent downregulation; ↑ represent upregulation.

miRNA	Source	Expression	Function	Ref.
hsa-miR-671-3p	Serum	↓	More than two-fold in PsA compare with PsV.	[21]
hsa-miR-151a-3p	Plasma	↑	Related to immune disorder and bone metabolic dysregulation.	[50]
hsa-miR-199a-5p				
hsa-miR-370-3p				
hsa-miR-589-5p				
hsa-miR-769-5p				
miR-1305	Serum	↑	Promoted keratinocyte proliferation and the secretion of CCL20 and IL-8.	[77]
miR-6785-5p		↓		
miR-381-3p	Keratinocytes	↑	Polarizating Th1 and Th17 in psoriasis.	[79]
miR-199a-3p	Serum	↑	Significantly up-regulating in patients with psoriasis versus healthy control.	[80]
let-7b-5p	Plasma	↓	As potential markers of PsA in patients with psoriasis.	[81]
miR-30e-5p				
miR-124-3p	Keratinocytes	↓	MiR124-3p/FGFR2 axis inhibits keratinocyte proliferation and migration.	[83]

## 5. Comprehensive Profiling of Extracellular Vesicle Cargo for Advancing Psoriasis Treatment Discovery

Recent research has delineated two primary therapeutic approaches utilizing mesenchymal stem cell-derived extracellular vesicles (MSCs-EVs) in the treatment of psoriasis. The first approach involves isolating EVs from cells and directly employing them as a therapeutic agent for psoriasis. The second strategy encompasses the encapsulation of therapeutic drugs within EVs, leveraging the vesicles as delivery vehicles to enhance the drug’s therapeutic efficacy.

### 5.1. Naive EVs

Naive EVs derived directly from cells, particularly targeting MSCs, have been explored for psoriasis treatment. MSCs have shown efficacy in the clinical management and research of inflammatory skin disorders over the past decade [86]. However, their use in preclinical studies [87] presents complex challenges and outcomes. Notable concerns include potential adverse effects, such as embolism and inefficient homing to target sites [21]. Consequently, EVs derived from MSCs are being considered as an alternative therapeutic strategy for psoriasis treatment.

In 2021, a pivotal study demonstrated that topically applied exosomes derived from MSCs could effectively attenuate levels of IL-17 and the terminal complement activation complex C5b-9 in IMQ-induced mice models of psoriasis (topical application at a dose of 20 µg per mouse) [88]. Topical application of MSC-derived EVs has been shown to inhibit complement activation in the stratum corneum, particularly by reducing the formation of C5b-9 complexes mediated by CD59. This intervention alleviates the accumulation of neutrophils within and beneath the stratum corneum, thereby mitigating the release of IL-17 through neutrophil extracellular traps (NETs). Subsequently, in 2022, a notable investigation utilized EVs obtained from MSCs modified via lentivirus-mediated gene transfection to overexpress PD-L1 (intravenous injection at a dose of 20 µg per mouse) [89]. This study suggested that such EVs could serve as a viable therapeutic approach for psoriasis, particularly due to the high expression of PD-1 in the autoimmune micro-environment of affected tissues. The specific use of PD-L1-enriched extracellular vesicles from lentivirus-transfected MSCs (MSC-sEVs-PD-L1) was found to significantly reduce the thickness of both skin and epidermis in IMQ-induced psoriasis models compared to controls. The mechanism of action involves MSC-sEVs-PD-L1 significantly targeting and repairing skin lesions through the PD-1/PD-L1 pathway, thereby suppressing the production of inflammatory immune cells at the lesion site. Furthermore, sEVs secreted from human umbilical cord-derived EVs have been recognized as a promising cell-free alternative and is considered a technology with promising potential for clinical translation [90]. Additionally, studies have indicated that exosomes from human umbilical cord MSCs (hucMSCs-Exo) can effectively lower the psoriasis area and severity index (PASI) scores and inhibit epidermal proliferation through subcutaneous injection (50 µg per mouse), while also significantly diminishing inflammatory factors, such as IL-17 and IL-23 [91]. hucMSCs-Exo improved the PASI scores and reduced inflammatory responses in psoriasis mice by regulating the expression of IL-23 and IL-17, as well as inhibiting the maturation and activation of dendritic cells (DCs).

In addition to mesenchymal stem cell-derived exosomes, EVs secreted from other cell types have also been explored for psoriasis treatment. Studies have demonstrated that treatment with sEVs derived from umbilical cord blood mononuclear cells (UCB-MNC-sEV) leads to a reduction in the levels of inflammatory mediators, including IL-6, IL-8, CXCL10, and COX-2 [92]. UCB-MNC-sEVs can act on various cell types, including keratinocytes, fibroblasts, monocytes, macrophages, and T cells, to reduce the expression of cytokines and mediators, such as TNF-α, COX2, IL-1β, IL-6, IFN-γ, CCL20, and CXCL8.

### 5.2. Engineering EVs

Novel pharmacological agents have been identified as potential avenues for alleviating psoriasis. However, challenges related to their instability and inefficient delivery have prompted the exploration of drug-loaded EVs as a delivery mechanism. One such agent, SO-210, has been recognized for its effectiveness in addressing the immune imbalance and pathological micro-environment in psoriasis. Due to its inherent instability and low delivery efficiency to the target cell, SO-210 has been encapsulated within sEVs, resulting in enhanced drug properties, suggesting MSC-sEVs as promising tools for the delivery of drugs (subcutaneous injection at a dose of 150 µg per mouse) [90]. Additionally, another study has reported that miR-124-3p, when delivered via EVs, can mitigate pathological and oxidative stress responses in IMQ-induced mouse models of psoriasis.

EVs have demonstrated multifunctional potential, not only as therapeutic agents themselves but as vehicles for drug delivery. To investigate the role of engineered EVs, JPH203 was encapsulated within ultraviolet B (UVB) radiation-stimulated EVs derived from keratinocytes, targeting psoriasis treatment. Interestingly, these EVs were found to decrease the level of IL-1RA following UVB irradiation. This observation suggests that EVs serve a dual function: not only as carriers of JPH203 but actively participating in the inhibition of the IL-1-mediated inflammatory cascade (subcutaneous injection at a dose of 0.625 mg JPH203 or 7.5 mg J@EVs per mouse). The mechanism of action involves J@EVs reducing keratinocyte hyperproliferation by inhibiting the mTOR signaling pathway and attenuating inflammatory responses by suppressing the NF-κB pathway. This indicates that extracellular vesicles not only serve as drug carriers but have the potential to suppress IL-1-mediated inflammatory cascades [93]. 

## 6. Implications for the Future

Over the past two decades, considerable advancements have been made in elucidating the therapeutic mechanisms and optimizing the treatment of psoriasis. Although conventional pharmacotherapy has been employed in managing this condition, the establishment of reliable biological diagnostic biomarkers could significantly enhance therapy efficacy by facilitating early-stage intervention. However, it is important to note that current therapeutic modalities for psoriasis are not without limitations in terms of convenience and permanency.

Emerging nanoscale biological vesicles, such as EVs, exhibit promising therapeutic potential for a diverse array of diseases. They play significant roles in various bodily fluids. Nevertheless, the current exploration and understanding of EVs remain in their infancy, indicating the necessity for more comprehensive research in this field. MicroRNAs, as highlighted previously, are emerging as a compelling category of small molecular biological entities. Owing to their unique properties, numerous studies have been directed toward their potential as long-term therapeutic agents to mitigate disease burden. However, the mechanisms governing microRNA sorting within EVs are still poorly understood, and the functional role of exosomal microRNAs in disease pathology remains largely conjectural. This uncertainty has led researchers to contemplate the broader applicability of this technology in clinical settings.

A recent review published in 2022 has shed light on the intricate protein machinery and nucleic acid sequence motifs associated with exosomal microRNAs, providing essential theoretical insights for enhancing drug loading efficiency in EVs [94]. In the realm of clinical trials, there has been noteworthy progress, particularly with the advancement of the first orally administered, drug-loaded EVs into phase II clinical trials for psoriasis patients. Concurrently, several studies have been examining the interplay between microorganisms and EVs. These investigations utilize the membranes of microorganisms as carriers, encapsulating therapeutic agents within EVs for efficient delivery to the patient’s body. This innovative method opens avenues for the development of advanced therapeutics for psoriasis. Additionally, the field of drug-loaded EVs presents considerable research potential. With the step-by-step exploration of natural medicines, these compounds are emerging as promising candidates for encapsulation within EVs.

EVs have emerged as promising tools in psoriasis research, both as biomarkers and therapeutic agents. Current evidence highlights their role in modulating inflammatory pathways and maintaining skin homeostasis. The potential to engineer EVs, particularly in combination with natural medicines, offers a novel approach to treatment, with the promise of fewer side effects and an improved safety profile compared to conventional therapies. Despite these advancements, key challenges remain. The heterogeneity of EVs complicates their standardization for clinical use, and the precise mechanisms underlying their therapeutic effects are not yet fully elucidated. Practical issues, such as efficient isolation, storage, and targeted delivery, further hinder their clinical translation. Large-scale, well-designed trials are urgently needed to validate their efficacy and safety.

Future research should focus on developing standardized protocols for EV characterization and exploring innovative engineering strategies to enhance their therapeutic specificity and functionality. Addressing these challenges will require close collaboration among researchers, clinicians, and industry partners. With continued effort, EV-based therapies have the potential to transform the management of psoriasis and advance the field of precision medicine.

## Data Availability

This article is a review of the existing literature, and it does not involve the generation or analysis of new datasets. All data referenced in this review are available from the cited original publications, which are publicly accessible through the respective journals or databases.

## Abbreviations

ApoMVs: apoptotic microvesicles; ApoEVs: apoptotic cell-derived EVs; ApoBDs: apoptotic bodies; EP: erythrodermic psoriasis; GA: gouty arthritis; hAD-MSCs: human adipose tissue-derived mesenchymal stem cells; HFSCs: hair follicle stem cell; ISEV: International Society of Extracellular Vesicles; LSEs: late-sorting endosomes; MSCs: mesenchymal stem cells; MVBs: multivesicular bodies; MSC-sEVs-PD-L1: PD-L1 extracellular vesicles derived from lentivirus-mediated gene transfection MSC; NTA: nanoparticle tracking analysis; PsA: psoriatic arthritis; PASI: psoriasis area and severity index; PsV: psoriasis vulgaris; PsC: cutaneous-only psoriasis; PR: pityriasis rosea; ROS: reactive oxygen species; RA: rheumatoid arthritis; sEVs: small EVs; TEM: transmission electron microscopy; UCB-MNC-sEV: umbilical cord blood mononuclear cell-derived sEV; UVB: ultraviolet B.
